# L-form bacteria, cell walls and the origins of life

**DOI:** 10.1098/rsob.120143

**Published:** 2013-01

**Authors:** Jeff Errington

**Affiliations:** The Centre for Bacterial Cell Biology, Newcastle University, Baddiley-Clark Building, Richardson Road, Newcastle upon Tyne NE2 4AX, UK

**Keywords:** L-forms, *Bacillus subtilis*, lipid vesicles, phylogenetics, origin of life

## Abstract

The peptidoglycan wall is a defining feature of bacterial cells and was probably already present in their last common ancestor. L-forms are bacterial variants that lack a cell wall and divide by a variety of processes involving membrane blebbing, tubulation, vesiculation and fission. Their unusual mode of proliferation provides a model for primitive cells and is reminiscent of recently developed *in vitro* vesicle reproduction processes. Invention of the cell wall may have underpinned the explosion of bacterial life on the Earth. Later innovations in cell envelope structure, particularly the emergence of the outer membrane of Gram-negative bacteria, possibly in an early endospore former, seem to have spurned further major evolutionary radiations. Comparative studies of bacterial cell envelope structure may help to resolve the early key steps in evolutionary development of the bacterial domain of life.

## 2. Uncertain evolutionary origins of the bacterial sub-kingdom

**Figure 1.**
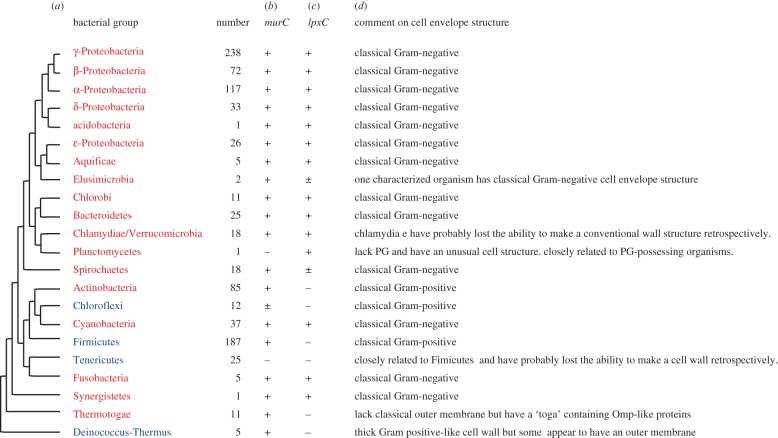
Outline phylogenetic tree of the bacteria and the distribution of key cell envelope features. (*a*) An outline phylogenetic tree for the bacteria, based on the major phylogenetic groups recognized by Wu *et al*. [3]. The detailed branching order is tentative for reasons outlined in the text. Groups traditionally described as Gram-negative and Gram-positive are indicated in red and blue text, respectively. (*b*,*c*) Distribution of genes required for PG (*b*) or Lipid A (*c*) synthesis, based on the output from STRING v. 9.0 [4] using *murC* and *lpxC* as markers. Similar results were obtained with other genes from the PG or the OM pathways (not shown). The number of organisms in each group are shown. Plus symbols (+) denote greater than 90% of organisms contained a likely gene homologue; plus or minus symbol (±), 50–70%; minus symbol (−), less than 5%. (*d*) Comments on current knowledge of cell envelope structures of various groups based mainly on information from Bergey's Manual of Systematic Bacteriology [5].

Bacteria occupy virtually every conceivable ecological niche on the planet and proliferate in vast numbers. The geological record and estimations from evolutionary clocks suggest that bacteria first appeared over 3 billion years ago [1]. Huge catalogues of rDNA sequences, supplemented by a rapidly increasing number of complete genome sequences, have provided crucial insights into the phylogenetic space occupied by bacteria, and led to the identification of about 20 major phyletic groups [2]. The new taxa expand on earlier groupings based largely on morphological and physiological traits. Figure 1*a* shows a typical tree [3], with most of the major recognized groups indicated. No attempt has been made here to put a scale on the length of the branches and the exact order of branching should be viewed as tentative. This is because at least two important factors confound attempts to define the root of the tree and the early order of branching. First, the majority of bacterial genes seem to have been subject to horizontal gene transfer and so generate inconsistent tree structures. Second, at the deepest phylogenetic levels the extent to which sequences are conserved disappears beneath the threshold at which statistical methods give reliable outcomes. Thus, how the major groups of bacteria emerged from the pre-cellular ‘primordial soup’ and began to differentiate from each other remains obscure [6–8].

## 3. The cell wall as a unifying trait among bacteria

Bacteria share a number of traits that distinguish them from the other major group of prokaryotes, the archaea and from eukaryotes. Most prominently, these include ‘information storage and processing’ functions: especially the DNA replication, transcription and translation machineries [9,10]. Another major feature thought to be unique to bacteria is the peptidoglycan (PG) cell wall. This important structure provides an external protective shell that serves several critical roles for those organisms that possess it. Traditionally, bacteria have been divided into two major subdivisions depending on whether they possess a second membrane (the outer membrane, OM) outside the PG wall. Gram-negatives or ‘diderms’ (red in figure 1*a*) have an OM, whereas Gram-positives (blue) lack this layer, often having a particularly thick PG layer instead. As summarized in figure 1*a,d*, there are a number of variations on this simple scheme, and envelope structure does not align completely with sequence-based taxonomies. Nevertheless, the cell wall is a crucial structure in almost all bacteria. Their growth and division are limited by the necessity to enlarge and then divide the wall. These processes are usually regulated spatially and temporally by important cytoskeletal proteins, MreB and FtsZ, that are distant relatives of actin and tubulin, respectively [11]. The biosynthetic pathways for PG precursor synthesis are well worked out and 10 or so genes encoding the key enzymes needed to make a functional wall can readily be identified. The levels of conservation of these various proteins and their respective genes are variable. However, several of the proteins appear to have excellent sequence conservation right across the bacterial sub-kingdom. Figure 1*b* shows an example of the conservation of a PG precursor synthesis gene, *murC*. Although a few groups of bacteria do not have a cell wall, in the well-characterized examples (e.g. *Tenericutes*, including *Mycoplasma*, *Phytoplasma*, etc.), it is clear that these organisms are closely related to major groups of bacteria that all possess a wall [12,13]. This strongly suggests that the exceptional organisms emerged by loss of the wall at some point in evolution.

On the basis of the near ubiquity and conserved synthesis and structure of the PG cell wall, it seems reasonable to assume that the last common ancestor (LCA) of all bacteria (if such a single ancestor ever existed) already had a wall. What, then, was the nature of cellular life before the invention of the wall? Recent discoveries with ‘L-form’ (cell wall-deficient) bacteria may provide clues as to the general cellular organization of the primitive progenitors of bacteria and especially how they proliferated.

## 4. Cell wall-deficient ‘L-form’ bacteria

**Figure 2.**
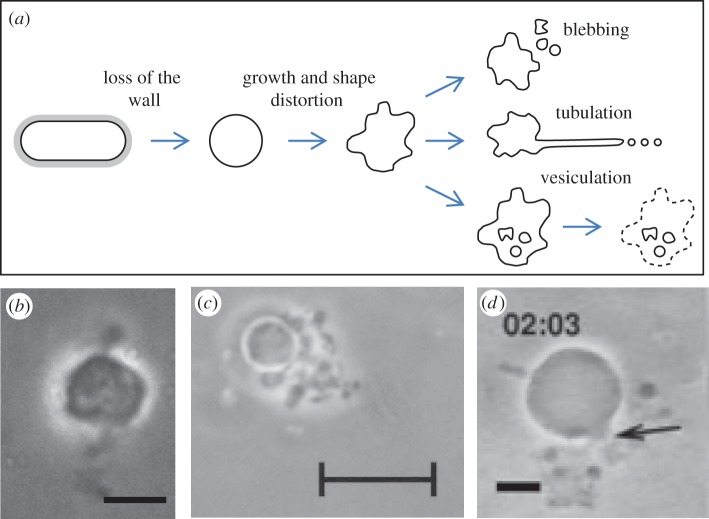
L-form proliferation and its similarity to *in vitro* vesicle replication. (*a*) Schematic of L-form proliferation based on observations with defined primary L-forms of *B. subtilis* [16] together with the vesiculation described by Dell'Era *et al.* [17]. (*b*–*d*) Comparison of L-form cells and replicating lipid vesicles. (*b*) A large *B. subtilis* L-form surrounded by recently generated progeny blebs [15]. (*c*) L-form-like cells from the urine of a Fanconi patient [18]. (*d*) *In vitro* proliferating lipid vesicle [19]. Scale bars: (*c*) 5 μm and (*d*,*e*) 10 μm.

Cell wall-deficient variants of bacteria that normally possess a wall have been described many times in the literature, since the original report of Klieneberger [14] nearly 80 years ago. We recently embarked on a molecular genetic analysis of the L-form state and showed that conversion of *Bacillus subtilis* into a form that can replicate reasonably efficiently in the absence of a cell wall (i.e. as an L-form) requires only two genetic changes [15] (R. Mercier, Y. Kawai & J. Errington 2012, unpublished data). Remarkably, despite the limited mutational changes required, the new L-form cells completely abandon the normally essential cell division machinery used by virtually all extant bacterial cells, and proliferate instead by a mechanism of membrane tubulation or blebbing (figure 2*a*). We showed, at least for *B. subtilis*, that this process is completely independent of the cell wall precursor synthetic pathway and the major cytoskeletal proteins, MreB and FtsZ. A recent report on *Listeria* L-forms highlighted a somewhat different process but which nevertheless again involves membrane dynamics (‘vesiculation’ in figure 2*a*) [17]. A wide range of bacteria are thought to be readily able to enter the L-form state, including both Gram-positive and -negative lineages [18]. Where their proliferation has been described, it generally resembles the mechanisms illustrated in figure 2. We have suggested that the membrane blebbing process used by *B. subtilis* L-forms represents an early mode of proliferation used by primitive cells before the invention of the cell wall [15,16]. The mechanism may have been retained by modern cells as a back-up process for use when cell wall synthesis is compromised. The risk to cell wall damage is probably ancient, given the widespread production of cell wall active antibiotics, such as β-lactams, glycopeptides and lipopeptides, by various ancient groups of bacteria [20,21].

## 5. Importance of membrane composition and dynamics for L-form proliferation

The finding that L-form proliferation is independent of cytoskeletal systems highlighted the question of its mechanism. Having established that blebbing and tubulation of L-forms was not dependent on known cytoskeletal systems we attempted to identify genes required specifically for this curious form of proliferation. The clearest mutant phenotype obtained, in which L-form proliferation was virtually abolished but growth of walled cells was unaffected, turned out to work by impairing branched chain fatty acid synthesis. Various lines of evidence point to this phenotype being manifested by a requirement for high membrane fluidity. Mutant cells with reduced membrane fluidity were able to grow in size and undergo some shape perturbations but they did not undertake the final membrane scission step needed to generate separate progeny cells [16]. The requirement for a particular state of membrane fluidity highlights the importance of the biophysical properties of membranes in L-form proliferation.

## 6. L-form-like proliferation under simple conditions *in vitro*

Meanwhile, as thoroughly reviewed recently by Briers *et al*. [22], *in vitro* experiments designed to reconstruct key steps required for the evolution of early forms of cellular life have generated outcomes remarkably reminiscent of the proliferation of L-form cells. One of the most important theoretical requirements for the evolution of life is a mechanism for encapsulating nucleic acids and the products of replication and gene expression [23]. A related requirement is for the cell envelope to replicate, while retaining and segregating the cell contents. Several recent studies have created plausible solutions to this problem by providing controlled lipid vesicle growth and fission under relatively simple *in vitro* conditions [19,24–28]. The theoretical basis for membrane vesicle fission is also well established [29,30]. In outline, shape perturbations leading to fission can be generated simply by increasing the vesicle surface area to volume ratio. A range of vesicles with different configuration and composition have been studied: unilamellar or multiple layered, and made up of simple fatty acids, as were probably abundant in the prebiotic era [31], through to more physiologically relevant phospholipid mixtures. Surface growth can be driven by ‘feeding’ with amphipathic molecules, such as fatty acids, either in solution or as micelles, which can intercalate or fuse with the vesicles to increase the surface area of the outermost leaflet or bilayer. Provided that the internal volume of the vesicle does not equilibrate rapidly, the mismatch between surface area and volume directly drives shape distortions that can lead to vesicle fission [19,25,28]. Similar outcomes can be generated by use of osmotic upshift to reduce interior volume at fixed surface area [24]. An example of the remarkable similarity in appearance between *in vitro* derived vesicle fission processes and L-form division is shown in figure 2*b–d*. Shape distortions and fission can also be promoted by intravesicular nanoparticles or macromolecules [26,27], suggesting a possible role for cell constituents, particularly the nucleoid, in promoting fission. Folding of the membrane around a nucleoid would directly promote the formation of a genetically viable progeny cell. The development of increasingly complex *in vitro* proliferation systems, more closely mimicking living cells, in parallel with further top-down dissection of L-form proliferation, appears an exciting area for future studies.

On the basis of the recent *in vitro* experiments and their similarity to L-form morphology and behaviour, it seems entirely plausible that membrane dynamics and purely biophysical processes could have allowed early cells to proliferate. It then follows that L-form proliferation could be a relic of this primitive mechanism originally used before the invention of the cell wall, but which remains accessible to modern cells.

## 7. L-form-like growth and proliferation would have supported massive genetic flux or horizontal gene transfer

Essentially, all modern organisms share a common genetic code, showing that some basic features of biological life became fixed relatively early. However, as discussed earlier, it is well known that archaea and eukaryotes differ significantly from the bacteria, especially in terms of the machinery used to replicate and express the information in DNA [9,10]. These differences are consistent with the notion that the mechanisms underlying various key cell functions were still in a state of flux when the early ancestors of the archaea and bacteria separated from each other.

We have observed L-forms to undergo spontaneous fusion, potentially generating heterokaryons or chimaeric genomes, under certain conditions (P. Domínguez-Cuevas & J. Errington 2012, unpublished data). Moreover, protoplasts (which are cells that are transiently converted into an L-form-like state but which do not normally undergo prolonged growth or division) have long been used by geneticists in fusion experiments to cross different bacterial strains, allowing the selection of recombinant genomes [32]. Assuming that ancient cellular organisms had a similar vesicular structure and used an L-form-like mode of proliferation, it seems likely that their primitive genomes would have been susceptible to continuous genetic flux. Fusion of vesicles containing dissimilar genomes would have allowed them to recombine or re-assort, and blebbing or tubulation would allow propagation and spread of the recombinant progeny. L-forms tend to have an unusually wide range of sizes and frequently contain multiple genomes [15,17]. The coincidence of multiple genomes in a single membrane bound vesicle would have facilitated genetic exchange.

## 8. Invention of the peptidoglycan wall and the bacterial radiation

It is generally assumed that a key step in the evolution of life must have involved a transition from an early acellular form of life in which horizontal gene transfer was rife, to more recognizably modern cellular organisms that could undergo tree-like evolutionary progression, leading to the elaboration of increasingly sophisticated forms. Woese [33] called this transition the ‘Darwinian Threshold’. However, there is presently no consensus view as to the possible basis for the emergence of stable cellular life forms. In line with a previous suggestion [34], I suggest that invention of the PG cell wall could have been a pivotal step in the evolution of bacteria.

Emergence of a wall would have conferred several important advantages to the genome that encoded it. First, the wall provides an external rigid or semi rigid ‘shell’ that offers protection from all kinds of damage, as well as the ability to explore and tolerate a much wider range of habitats, particularly of differing osmolarities, than their simple membrane-bound predecessors. Second, the wall would provide much tighter control of cell size, shape, division, growth orientation, etc., especially in conjunction with a cytoskeleton to provide spatial regulation. Certainly, walled *B. subtilis* cells grow much faster than their isogenic L-form counterparts [15]. Third, the wall would almost certainly have had a dramatic effect on genome stability, protecting cells from the rampant horizontal gene transfer that probably occurred in cells with a naked cytoplasmic membrane. This would in turn have allowed a much more sophisticated integration of the genetic operating system (replication, transcription and translation) with the informational genes specifying the day-to-day life of the organism and its interactions with the environment.

**Figure 3.**
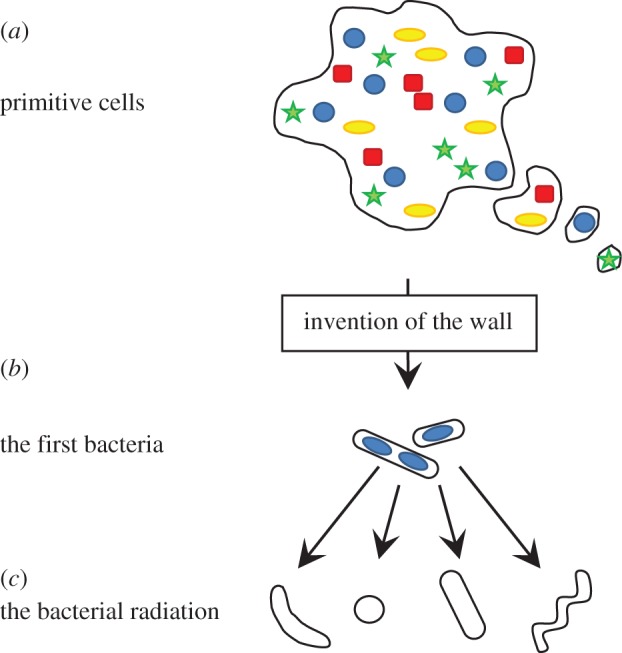
Pivotal role for the cell wall in the bacterial radiation. (*a*) Schematic of a primitive cell bounded by a single lipid bilayer. Objects in different colours and shapes represent distinct separately evolving proto-genomes. The cells proliferate by tubulation or blebbing, similarly to L-forms. Such cells would have undergone frequent fusion and fission events, resulting in rampant horizontal gene transfer. The cells would have been fragile and overall growth slow. (*b*) Invention of an early form of the cell wall would have led to early forms of true bacteria. The presence of the wall would have crystallized the genome from which it was encoded by largely preventing horizontal gene transfer. Polarized growth of the wall would have enhanced the efficiency of growth and division, as well as chromosome replication and segregation. (*c*) Rapid and efficient growth, together with a tough protective layer would have enabled effective exploration of novel niches and the evolution of many new forms.

The advantages offered by invention of the wall could have enabled an explosion of successful new forms all sharing the PG wall system but featuring increasingly elaborate variations able to explore and exploit new environments (figure 3).

## 9. Emergence of archaea by invention of a different cell envelope structure(s)?

It is well known that archaea tend to have different cell envelope structures to bacteria [35]. Informatic analysis of the large number of archaeal genomes now available confirms that they completely lack the multi-step biosynthetic pathway for PG precursors (J. Errington 2012, unpublished data). Unlike bacteria, archaea display a variety of different protective surface layers, most commonly, a paracrystalline proteinaceous shell called an S-layer [35]. Several families of archaea have a PG-like layer called pseudomurein, but the precursors for assembly of this kind of wall are synthesized by a completely different enzymatic pathway from that of PG. In the light of the above discussion, it seems possible that the archaea emerged from the ‘protocell soup’ by invention of one or more different cell envelope structures. The genome captured by the successful enveloped archaeal progenitors had a related but distinct operating system, explaining the differences in replication, transcription and translational apparatus between bacteria and archaea.

## 10. Evolutionary development of the bacterial cell envelope

The idea that bacteria underwent a major radiation following the invention of the wall predicts that it should be possible to discern a pathway of emergence of the major modern bacterial groups, in parallel with an elaboration of wall structures. Unfortunately, sequence-based homology detection methods have not yet provided a robust and reliable means of discerning branching order at the deepest levels of bacterial evolution [2]. However, Jensen and co-workers [36] recently provided a major new insight into this problem by discovery of a likely origin, perhaps for all of the Gram-negative bacteria, within the *Veillonellacea* group of the *Firmicutes* (essentially the major group of low G + C Gram-positive bacteria). The relationship between the Gram-negative and Gram-positive bacteria has been the matter of much speculation and discussion. Some have viewed the diderms as likely to have evolved from the monoderms by evolution of a more complex cell envelope [37]. Gupta suggested that antibiotic selection provided the driving force for development of this additional protective layer. By contrast, Lake [38] suggested that the OM arose following an ancient endosymbiotic fusion of actinobacterial and clostridial forms. A diametrically opposing view is that the diderm form was ancestral and that monoderms emerged by loss of the OM [39]. Schemes such as the one shown in figure 1*a* do not help in distinguishing between the two models because the Gram-positive and -negative groups appear interspersed. But as explained earlier, this could be due to lack of statistical resolution in the deeper branches of the tree.

**Figure 4.**
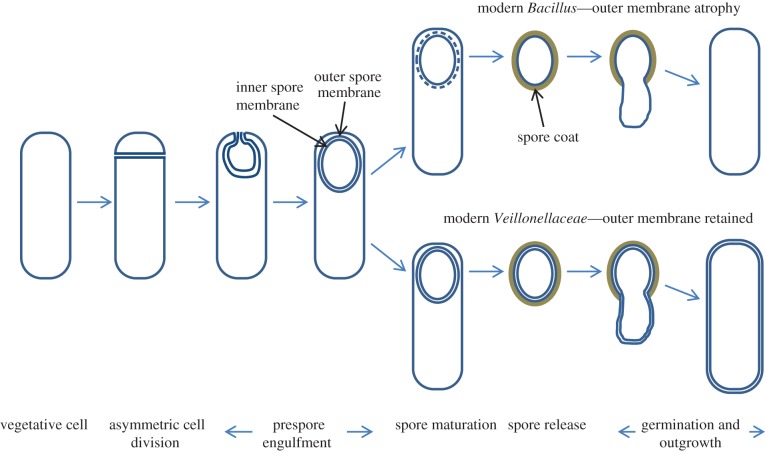
Evolution of the Gram-negative OM via endospore formation. Key steps in the general sporulation process are labelled below. For simplicity, growth and early steps of sporulation of modern *Veillonaceae* in the diderm state are not shown.

**Figure 5.**
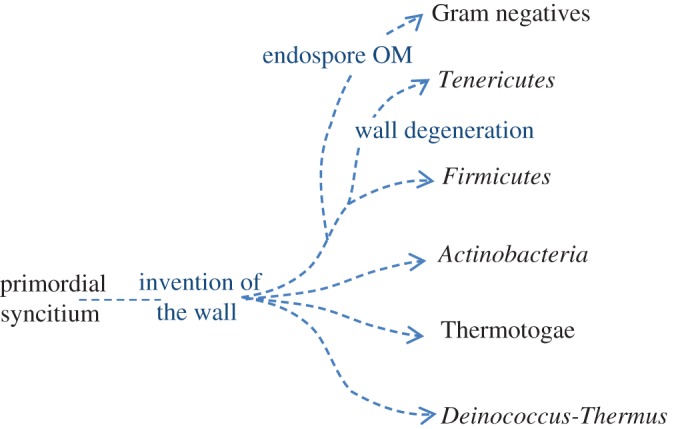
Revised scheme for the evolutionary divergence of the bacteria. Invention of the PG cell wall resulted in the earliest bacteria with a simple monoderm structure. The evolutionary relationships between modern *Firmicutes*, *Actinobacteria*, *Chloroflexi* and the *Deinococcus-Thermus* group remain unclear. The major Gram-negative phyla are now suggested to have emerged from within the ancestors of the *Firmicutes* by retention of the endospore OM. The *Tenericutes* probably emerged from the same ancient group by complete loss of the wall.

The main problem with the first of the above scenarios was the question of how the OM could have been invented. A solution to this problem, strongly supporting the likelihood of a monoderm → diderm order of emergence, was provided by recognition that even though the *Veillonellacea* clearly belong to the ancient *Firmicutes* lineage, they are Gram-negative in organization. Furthermore, their OM originates in the OM of the endospore, which is a specialized cell-type characteristic of the *Firmicutes*. Figure 4 shows the well-characterized steps of endospore formation, as largely worked out through decades of research on *B. subtilis* and its relatives. Engulfment of the prespore by the mother cell leads to a ‘cell within a cell’ and in particular, generates a prespore with a double membrane. In *B. subtilis* and many other spore formers, the OM disappears during spore development and is not visible when the spore eventually germinates. However, in the *Veillonellacea* or at least some members of this group (specifically, *Acetonema longum* [36]), the OM remains and is retained through to spore outgrowth and beyond, as normal vegetative growth is restored. Detailed informatic searches support the idea that the *Acetonema* group (*Veillonellacea*) are truly core members of the phylum *Firmicutes*. If this idea is correct it has profound implications for the evolutionary development of bacteria because the whole diderm radiation—including many or all of the red ‘Gram-negative’ bacteria listed in figure 1*a* would be descendants of a branch in the *Firmicutes* lineage (figure 5). The root of the whole bacterial lineage would then lie somewhere in the ancestors of a relatively small group of monoderm bacteria.

Molecular markers specific for OM biogenesis are limited because many of the proteins, e.g. Omps, show poor sequence conservation. The *lpxC* gene presently seems to be the best surrogate [36]. It is a well-conserved cytoplasmic protein carrying out a key specific step in the synthesis of LipidA, the major lipid component of the OMs of many diderm bacteria. As shown in figure 1*c*, *lpxC* tracks well with the diderm state. Included are several major subgroups of organisms of previously uncertain origin: *Cyanobacteria, Chlorobi, Bacteroides, Planctomyces* and *Chlamydia*. We cannot rule out the alternative hypothesis, that these organisms derived *lpxC* and multiple genes needed to make an OM by lateral gene transfer, but this seems unlikely.

The explosion of genome sequencing is providing a gold mine of information on the diversity and versatility of the bacteria. Nevertheless, there are still major gaps in our knowledge of how the bacterial (and archaeal) groups emerged and then diverged. Sequence-based analytical methods are still inadequate for defining the early steps in the bacterial radiation. Several groups of bacteria that may be representative of pivotal steps in the evolutionary sequence are poorly characterized. Comparative studies of the structure and synthesis of the cell envelopes of a wider range of organisms may provide important clues about the evolutionary development of the bacteria. *Thermotoga* provides an interesting example. Its defining feature is a loose outer layer called a toga. This was thought to be related in some way to the OM of diderm bacteria. However, as shown in figure 1*a*, these organisms lack an *lpxC* gene and therefore can probably not synthesize LipidA. Nevertheless, the toga was recently shown to contain classical Omp-like proteins [40], consistent with these organisms having a diderm ancestry. It seems likely that further detailed studies of the cell envelopes of deeply rooted bacterial groups will provide important insights into the early evolutionary history of the bacteria.

## 11. Acknowledgements

I thank Martin Embley and Kenn Gerdes for insightful comments on the manuscript. Work on L-form bacteria in my laboratory is funded by a European Research Council Senior Investigator Award.

## References

[1] Sleep NH Bird DK Pope EC 2011 Serpentinite and the dawn of life. Phil. Trans. R. Soc. B 366, 2857–2869 10.1098/rstb.2011.0129 (doi:10.1098/rstb.2011.0129) 21930576 PMC3158911

[2] Pace NR 2009 Mapping the tree of life: progress and prospects. Microbiol. Mol. Biol. Rev. 73, 565–576 10.1128/mmbr.00033-09 (doi:10.1128/mmbr.00033-09) 19946133 PMC2786576

[3] Wu D 2009 A phylogeny-driven genomic encyclopaedia of Bacteria and Archaea. Nature 462, 1056–1060 10.1038/nature08656 (doi:10.1038/nature08656) 20033048 PMC3073058

[4] Szklarczyk D 2011 The STRING database in 2011: functional interaction networks of proteins, globally integrated and scored. Nucleic Acids Res. 39, D561–D568 10.1093/nar/gkq973 (doi:10.1093/nar/gkq973) 21045058 PMC3013807

[5] Bergey's Manual Trust 2012 Bergey's manual of systematic biology, *2nd edn*. New York, NY: Springer passim

[6] Doolittle WF 1999 Phylogenetic classification and the universal tree. Science 284, 2124–2129 10.1126/science.284.5423.2124 (doi:10.1126/science.284.5423.2124) 10381871

[7] Dagan T Martin W 2006 The tree of one percent. Genome Biol. 7, 118 10.1186/gb-2006-7-10-118 (doi:10.1186/gb-2006-7-10-118) 17081279 PMC1794558

[8] Puigbo P Wolf YI Koonin EV 2009 Search for a ‘Tree of Life’ in the thicket of the phylogenetic forest. J. Biol. 8, 59 10.1186/jbiol159 (doi:10.1186/jbiol159) 19594957 PMC2737373

[9] Olsen GJ Woese CR 1996 Lessons from an Archaeal genome: what are we learning from *Methanococcus jannaschii*? Trends Genet. 12, 377–379 10.1016/0168-9525(96)30092-9 (doi:10.1016/0168-9525(96)30092-9) 8909123

[10] Rivera MC Jain R Moore JE Lake JA 1998 Genomic evidence for two functionally distinct gene classes. Proc. Natl Acad. Sci. USA 95, 6239–6244 10.1073/pnas.95.11.6239 (doi:10.1073/pnas.95.11.6239) 9600949 PMC27643

[11] Typas A Banzhaf M Gross CA Vollmer W 2012 From the regulation of peptidoglycan synthesis to bacterial growth and morphology. Nat. Rev. Microbiol. 10, 123–136 10.1038/nrmicro2677 (doi:10.1038/nrmicro2677) 22203377 PMC5433867

[12] McInerney JO Martin WF Koonin EV Allen JF Galperin MY Lane N Archibald JM Embley TM 2011 Planctomycetes and eukaryotes: a case of analogy not homology. Bioessays 33, 810–817 10.1002/bies.201100045 (doi:10.1002/bies.201100045) 21858844 PMC3795523

[13] Trachtenberg S 1998 Mollicutes-wall-less bacteria with internal cytoskeletons. J. Struct. Biol. 124, 244–256 10.1006/jsbi.1998.4063 (doi:10.1006/jsbi.1998.4063) 10049810

[14] Klieneberger E 1935 The natural occurrence of pleuropneumonia-like organisms in apparent symbiosis with *Streptobacillus moniliformis* and other bacteria. J. Pathol. Bacteriol. 40, 93–105 10.1002/path.1700400108 (doi:10.1002/path.1700400108)

[15] Leaver M Domínguez-Cuevas P Coxhead JM Daniel RA Errington J 2009 Life without a wall or division machine in *Bacillus subtilis*. Nature 457, 849–853 10.1038/nature07742 (doi:10.1038/nature07742) 19212404

[16] Mercier R Domínguez-Cuevas P Errington J 2012 Crucial role for membrane fluidity in proliferation of primitive cells. Cell Reports 1, 417–423 10.1016/j.celrep.2012.03.008 (doi:10.1016/j.celrep.2012.03.008) 22832271

[17] Dell'Era S Buchrieser C Couve E Schnell B Briers Y Schuppler M Loessner MJ 2009 *Listeria monocytogenes* L-forms respond to cell wall deficiency by modifying gene expression and the mode of division. Mol. Microbiol. 73, 306–322 10.1111/j.1365-2958.2009.06774.x (doi:10.1111/j.1365-2958.2009.06774.x) 19555455

[18] Domingue GJ Sr Woody HB 1997 Bacterial persistence and expression of disease. Clin. Microbiol. Rev. 10, 320–344 9105757 10.1128/cmr.10.2.320 PMC172922

[19] Peterlin P Arrigler V Kogej K Svetina S Walde P 2009 Growth and shape transformations of giant phospholipid vesicles upon interaction with an aqueous oleic acid suspension. Chem. Phys. Lipids 159, 67–76 10.1016/j.chemphyslip.2009.03.005 (doi:10.1016/j.chemphyslip.2009.03.005) 19477312

[20] Goodfellow M Fiedler HP 2010 A guide to successful bioprospecting: informed by actinobacterial systematics. Antonie Van Leeuwenhoek 98, 119–142 10.1007/s10482-010-9460-2 (doi:10.1007/s10482-010-9460-2) 20582471

[21] Gupta RS 2011 Origin of diderm (Gram-negative) bacteria: antibiotic selection pressure rather than endosymbiosis likely led to the evolution of bacterial cells with two membranes. Antonie Van Leeuwenhoek 100, 171–182 10.1007/s10482-011-9616-8 (doi:10.1007/s10482-011-9616-8) 21717204 PMC3133647

[22] Briers Y Walde P Schuppler M Loessner MJ 2012 How did bacterial ancestors reproduce? Lessons from L-form cells and giant lipid vesicles: multiplication similarities between lipid vesicles and L-form bacteria. Bioessays 34, 1078–1084 10.1002/bies.201200080 (doi:10.1002/bies.201200080) 23108858

[23] Budin I Szostak JW 2010 Expanding roles for diverse physical phenomena during the origin of life. Annu. Rev. Biophys. 39, 245–263 10.1146/annurev.biophys.050708.133753 (doi:10.1146/annurev.biophys.050708.133753) 20192779 PMC4992673

[24] Andes-Koback M Keating CD 2011 Complete budding and asymmetric division of primitive model cells to produce daughter vesicles with different interior and membrane compositions. J. Am. Chem. Soc. 133, 9545–9555 10.1021/ja202406v (doi:10.1021/ja202406v) 21591721 PMC3115689

[25] Inaoka Y Yamazaki M 2007 Vesicle fission of giant unilamellar vesicles of liquid-ordered-phase membranes induced by amphiphiles with a single long hydrocarbon chain. Langmuir 23, 720–728 10.1021/la062078k (doi:10.1021/la062078k) 17209626

[26] Terasawa H Nishimura K Suzuki H Matsuura T Yomo T 2012 Coupling of the fusion and budding of giant phospholipid vesicles containing macromolecules. Proc. Natl Acad. Sci. USA 109, 5942–5947 10.1073/pnas.1120327109 (doi:10.1073/pnas.1120327109) 22474340 PMC3340996

[27] Yu Y Granick S 2009 Pearling of lipid vesicles induced by nanoparticles. J. Am. Chem. Soc. 131, 14 158–14 159 10.1021/ja905900h (doi:10.1021/ja905900h) 19775107

[28] Zhu TF Szostak JW 2009 Coupled growth and division of model protocell membranes. J. Am. Chem. Soc. 131, 5705–5713 10.1021/ja900919c (doi:10.1021/ja900919c) 19323552 PMC2669828

[29] Svetina S 2009 Vesicle budding and the origin of cellular life. Chemphyschem 10, 2769–2776 10.1002/cphc.200900577 (doi:10.1002/cphc.200900577) 19774545

[30] Svetina S Zeks B 2002 Shape behavior of lipid vesicles as the basis of some cellular processes. Anat. Rec. 268, 215–225 10.1002/ar.10156 (doi:10.1002/ar.10156) 12382320

[31] Monnard PA Deamer DW 2002 Membrane self-assembly processes: steps toward the first cellular life. Anat. Rec. 268, 196–207 10.1002/ar.10154 (doi:10.1002/ar.10154) 12382318

[32] Hopwood DA Wright HM Bibb MJ Cohen SN 1977 Genetic recombination through protoplast fusion in *Streptomyces*. Nature 268, 171–174 10.1038/268171a0 (doi:10.1038/268171a0) 593313

[33] Woese CR 2002 On the evolution of cells. Proc. Natl Acad. Sci. USA 99, 8742–8747 10.1073/pnas.132266999 (doi:10.1073/pnas.132266999) 12077305 PMC124369

[34] Kandler O 1994 Cell wall biochemistry in Archaea and its phylogenetic implications. J. Biol. Phys. 20, 165–169 10.1007/bf00700433 (doi:10.1007/bf00700433)

[35] Albers SV Meyer BH 2011 The archaeal cell envelope. Nat. Rev. Microbiol. 9, 414–426 10.1038/nrmicro2576 (doi:10.1038/nrmicro2576) 21572458

[36] Tocheva EI Matson EG Morris DM Moussavi F Leadbetter JR Jensen GJ 2011 Peptidoglycan remodeling and conversion of an inner membrane into an outer membrane during sporulation. Cell 146, 799–812 10.1016/j.cell.2011.07.029 (doi:10.1016/j.cell.2011.07.029) 21884938 PMC3176627

[37] Koch AL 2003 Were Gram-positive rods the first bacteria?. Trends Microbiol. 11, 166–170 10.1016/S0966-842X(03)00063-5 (doi:10.1016/S0966-842X(03)00063-5) 12706994

[38] Lake JA 2009 Evidence for an early prokaryotic endosymbiosis. Nature 460, 967–971 10.1038/nature08183 (doi:10.1038/nature08183) 19693078

[39] Cavalier-Smith T 2002 The neomuran origin of archaebacteria, the negibacterial root of the universal tree and bacterial megaclassification. Int. J. Syst. Evol. Microbiol. 52, 7–76 11837318 10.1099/00207713-52-1-7

[40] Petrus AK Swithers KS Ranjit C Wu S Brewer HM Gogarten JP Pasa-Tolic L Noll KM 2012 Genes for the major structural components of Thermotogales species’ togas revealed by proteomic and evolutionary analyses of OmpA and OmpB homologs. PLoS ONE 7, e40236 10.1371/journal.pone.0040236 (doi:10.1371/journal.pone.0040236) 22768259 PMC3387000

